# Takotsubo Cardiomyopathy Coexisting with Acute Pericarditis and Myocardial Bridge

**DOI:** 10.1155/2016/5189741

**Published:** 2016-06-29

**Authors:** Seyed Hashem Sezavar, Neda Toofaninejad, Shokoufeh Hajsadeghi, Hassan Riahi Beni, Reza Ghanavati, Marjan Hajahmadi, Morteza Hassanzadeh

**Affiliations:** ^1^Department of Cardiology, Rasoul-e-Akram Hospital, Iran University of Medical Sciences, Tehran 14456 13131, Iran; ^2^Department of Cardiology, Qom University of Medical Sciences, Qom, Iran; ^3^Department of Internal Medicine, Rasoul-e-Akram Hospital, Iran University of Medical Sciences, Tehran, Iran

## Abstract

Takotsubo cardiomyopathy (TCM) is a stress-induced cardiomyopathy that occurs primarily in postmenopausal women. It mimics clinical picture of acute coronary syndrome with nonobstructive coronary arteries and a characteristic transient left (or bi-) ventricular apical ballooning at angiography. The exact pathogenesis of TCM is not well recognized. Hereby we present an unusual case of TCM that presents with signs and symptoms of acute pericarditis and was also found to have a coexisting coronary muscle bridge on coronary angiography. We discuss the impact of these associations in better understanding of the pathogenesis of TCM.

## 1. Introduction

Takotsubo cardiomyopathy (TCM) or transient left ventricular apical ballooning syndrome is a novel type of cardiomyopathy that was first described in 1990 as a differential diagnosis for acute coronary syndrome in the absence of obstructive coronary artery disease [1]. It predominantly affects postmenopausal women and is often preceded by a physical or emotional stress [2, 3]. Although the exact pathogenesis of TCM is not well recognized, the role of excessive catecholamine release has been proposed to act via some possible mechanisms such as coronary artery vasospasm (close to mechanism of Prinzmetal's angina [4]) and/or regional myocardial dysfunction with contraction band necrosis (similar to mechanism of electrocardiographic changes after subarachnoid hemorrhage [5]).

Hereby we present an unusual case of TCM that presents with signs and symptoms of acute pericarditis and was found to have a coexisting coronary muscle bridge on coronary angiography (CAG), associations that might have an impact on better understanding of the pathogenesis of TCM.

## 2. Case Report

**Figure 1:**
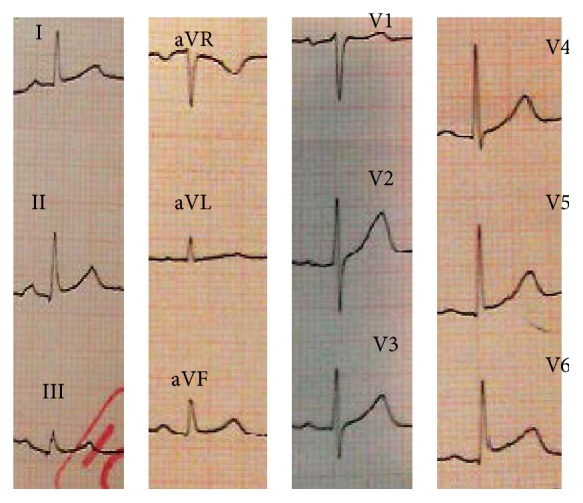
Initial electrocardiogram of the patient at the admission time.

**Figure 2:**
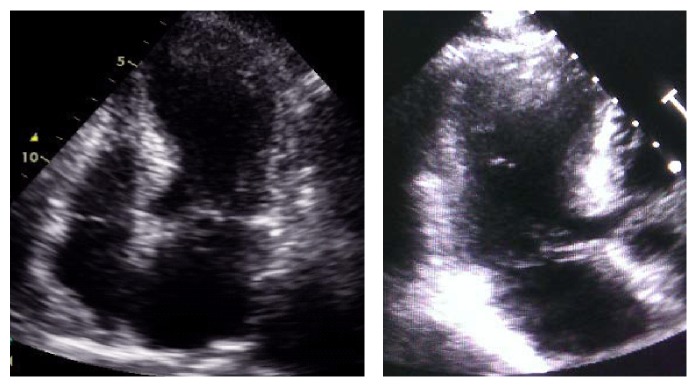
Echocardiography showing apical hypokinesis with preserved basal function.

A 42-year-old Iranian woman was brought to the emergency department with a sharp, persistent pleuritic chest pain beginning after an emotional stress. She had neither remarkable past medical history for coronary risk factors nor any relevant past interventions. Physical examination revealed a heart rate of 85 beats per minute, blood pressure of 120/80 mmHg, and normal S1 and S2 with S3 gallop as well as a triphasic friction rub (one systolic and two diastolic sounds) at cardiac auscultation. An initial electrocardiogram (ECG) demonstrated 1-2 mm ST elevation in leads I, II, III, aVF, and V4–V6, as well as ST depression and PR elevation at aVR (Figure 1). A transthoracic echocardiography (TTE) on admission showed an apical akinesia with a preserved basal function, a mild pericardial effusion, an ejection fraction of 40%, and a moderate mitral regurgitation (Figure 2).

**Figure 3:**
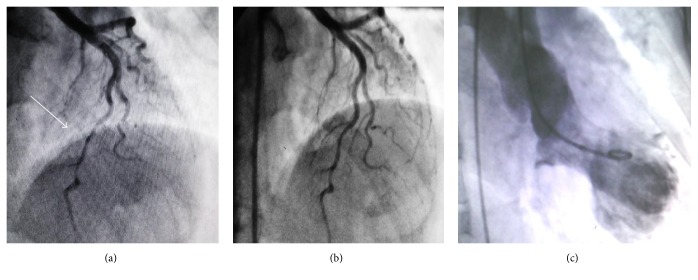
Coronary angiography revealed severe muscle bridge at mid portion of left anterior descending artery during systole (a) and diastole (b). Left ventriculography showed systolic ballooning of the apex consistent with Takotsubo cardiomyopathy (c).

After finding wall motion abnormality on TTE and because of persistent chest pain, despite the initial clinical diagnosis of acute pericarditis, the patient was immediately transferred to the cardiac catheter laboratory in order to look for any coronary artery disease requiring intervention. CAG demonstrated a severe muscle bridge at mid portion of the left anterior descending artery (LAD) and otherwise normal coronary arteries (Figure 3); left ventriculography showed characteristic systolic ballooning of the apex in favor of TCM (Figure 3). Result of the laboratory tests revealed mildly elevated serum levels of cardiac enzymes: creatine kinase MB = 30 ng/mL (normal range < 25 ng/mL) and troponin I = 0.89 ng/mL (normal range < 0.01).

**Figure 4:**
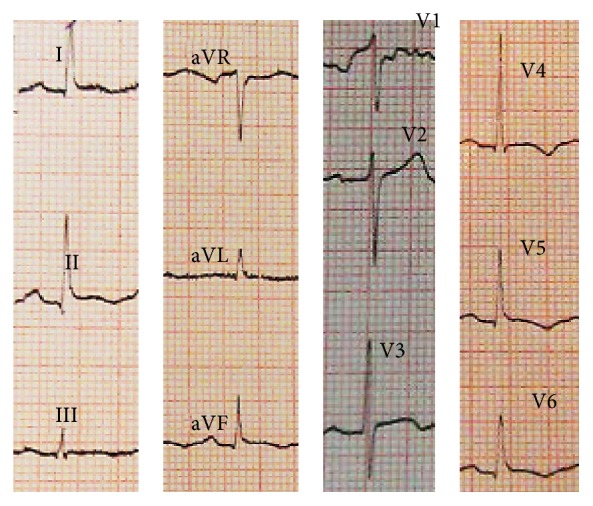
The electrocardiogram at 2nd day of admission revealing “mild” T-wave inversion at leads I, II, III, aVL, aVF, and V4–V6.

**Figure 5:**
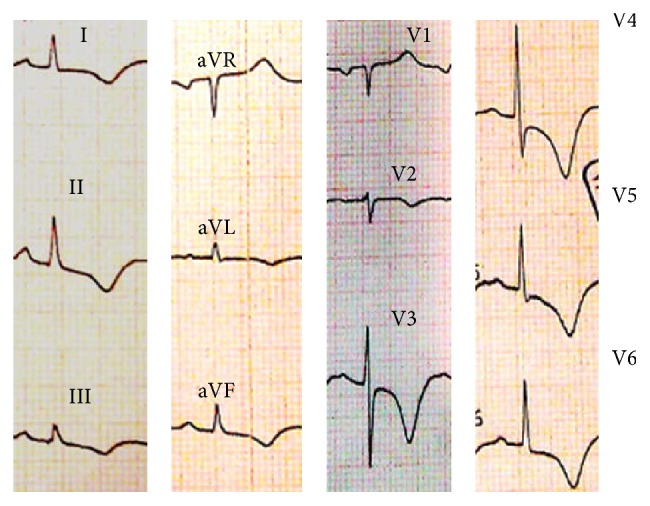
The electrocardiogram at 3rd day of admission revealing “deep” T-wave inversion at leads I, II, III, aVL, aVF, and V2–V6.

The patient was treated with an angiotensin-converting enzyme inhibitor (captopril 6.25 mg three times per day), a diuretic (furosemide 20 mg three times per day), and a beta-blocker (carvedilol 3.125 two times per day). She also received aspirin for 4 days at high enough doses for treating pericarditis (650 mg three times per day). On serial ECG mild T-wave inversion was found in leads I, II, III, aVL, aVF, and V4–V6 at 2nd day of admission which then became deeper at 3rd day (Figures 4 and 5).

The patient's general condition considerably improved over the next few days and her chest pain was completely resolved. One week later, a subsequent TTE at discharge day revealed an improved left ventricular ejection fraction of 50–55% with no apical ballooning, no significant mitral regurgitation, and no pericardial effusion.

## 3. Discussion

In the present case, the fairly sudden onset of a sharp and pleuritic chest pain, the characteristic three-phasic pericardial friction rub, the mild pericardial effusion on TTE, and the significant clinical response to high-dose aspirin all are in favor of acute pericarditis. On the other hand, such features as the clear history of an emotional stress before the onset of chest pain, diffuse ST elevation on ECG with the patent coronary arteries at CAG, and the characteristic apical ballooning at TTE with spontaneous recovery are suggesting features of TCM. Finally, detection of coronary muscle bridge at CAG adds a third diagnostic issue to this patient's presentation.

After the first formal description of TCM in 1990 in Japan [1], several patients have been reported with this diagnosis worldwide. Although it has been described to predominantly affect postmenopausal women with a clinical presentation similar to acute coronary syndrome [3], cases of TCM at a different age group [6–8] (even at childhood [9]) or with unusual presentations [10–12] have also been reported. Our patient, a woman at premenopausal age which is not typical for TCM, presented with the clinical picture of acute pericarditis. Association of TCM with pericarditis has been reported in at least 8 reports among which at 3 cases pericarditis presented as a complication of TCM [13–15] and at the other 5 ones pericarditis preceded the presentation of TCM [9, 16–19]. In the former cases, regional inflammatory myocarditis, which has been previously reported both at endomyocardial biopsy specimens [20] and at magnetic resonance imaging (MRI) of TCM patients [21], has been hypothesized to be extended to the overlying pericardium and then is clinically presented as pericarditis [22]. The role of intense catecholamine release as a result of severe pericarditis-induced chest pain, however, has been supposed to describe pericarditis as a trigger for TCM in the latter cases [22]. In our case, the scenario began with an emotional stress which led to an initial clinical picture of acute pericarditis and then was established as TCM. History of a preceding major stress, similar to our patient, was described in only one out of those 5 cases that presented with pericarditis and then developed TCM. With focusing on this unique presentation, TCM presents as acute pericarditis without the history of recent febrile illness but instead with the history of a major stressful event, an intermediary mechanism for combined pericarditis, and TCM in this case would be considering the whole scenario as a variant of “perimyocarditis” [23]. It means that, considering the inflammatory hypothesis for TCM, a clinicopathophysiological spectrum of TCM patients could be hypothesized: those TCM cases with the inflammation mostly limited to myocytes with minimal or no pericardial involvement are at one end of the spectrum, and those in which the pericardial component of the inflammation is more dominant are at the other end. Sequential ECG might help differentiate the latter cases from those with simple pericarditis: a rapid development of T-wave inversion after the phase of ST-segment elevation (as in our case) is in favor of TCM [24]. However, patients with pure pericarditis have slower progression of T-wave inversion.

According to the proposed diagnostic criteria of Mayo Clinic, absence of myocarditis is needed to confirm the diagnosis of TCM [25]. Moreover, using cardiac MRI, Eitel et al. [26] demonstrated that 8 out of 59 patients (13.6%) with apical ballooning at CAG had a final diagnosis of myocarditis. Considering these points and the fact that we did not perform cardiac MRI for the patient, one might be concerned about a neglected diagnosis of myocarditis rather than “real” TCM. However, the rather acute onset of chest pain with a prominent pleuritic pattern, the disproportionate elevation of cardiac enzymes (to the extensive ECG changes), and the rapid resolution of the disease all make it less likely to consider myocarditis as the final diagnosis of this patient.

Another interesting point in this case was coexistence of myocardial bridge, a congenital coronary anomaly characterized by systolic compression of a part of an epicardial vessel by a segment of overlying myocardium, and TCM. Association of myocardial bridge and TCM has been reported in some case reports and case series [27–30], which suggest that myocardial bridging of the LAD is a frequent finding in patients with apical ballooning syndrome. Actually, the high circulating level of catecholamines has been considered to uncover myocardial bridging in some patients with an apical ballooning syndrome and to make it angiographically evident [30]. However, myocardial bridge could also be a simple anatomical variant and is not necessarily associated with TCM. In such patients, by repeating CAG after a certain period of time, it can be ascertained if the myocardial bridging is still present (and is simply a coincidental finding) or not.

In conclusion, to the best of our knowledge, this is the first report of coexistence of myocardial bridging, TCM, and acute pericarditis in a single patient, which may suggest a potential role of both myocardial bridging and acute pericarditis in the pathogenesis of TCM.
